# Treatment of municipal wastewater by vertical subsurface flow constructed wetland: Data collection on removal efficiency using Phragmites Australis and Cyperus Papyrus

**DOI:** 10.1016/j.dib.2020.105584

**Published:** 2020-04-18

**Authors:** Fernando García-Ávila

**Affiliations:** Facultad de Ciencias Químicas, Universidad de Cuenca, Cuenca, Ecuador

**Keywords:** Constructed wetland, Macrophytes, Nutrients, Wastewater treatment, Phragmites Australis, Cyperus Papyrus

## Abstract

The data of this document highlights a comparative analysis between the Phragmites Australis and Cyperus Papyrus planted in vertical subsurface flow constructed wetland (VSSFCW) independently implemented at pilot scale for the treatment of domestic wastewater. At the exit of the primary lagoon a pipe was installed to feed a constant flow of 0.6 m^3^/day to each of the two constructed wetlands. Each unit had a retention time of 1.12 days and a hydraulic load rate of 0.2 m/day. To evaluate the efficiency of the treatment, physical, chemical and biological parameters were monitored for three consecutive months. Water samples in the influent and effluent of each experimental wetland were analyzed. At the end of the monitoring, Cyperus Papyrus had a better removal of total phosphorus (50%), ammoniacal nitrogen (69.69%), biochemical oxygen demand (69.87%), chemical oxygen demand (80.69%), total coliforms (98.08%) and fecal coliforms (95.61%). Meanwhile, Phragmites Australis eliminated total solids better (62.85%). These data may be useful for comparative purposes on domestic wastewater treatment using Phragmites Australis and Cyperus Papyrus planted in VSSFCW.

Specifications table

**Table:** 

Subject area	Environmental Engineering
More specific subject area	Wastewater treatment
Type of data	Table and figure
How data was acquired	A fortnightly sampling was carried out for three months. Water samples were collected at three points: at the entrance to the wetlands (the wastewater that entered was the same for the two wetlands) and a sampling point at the exit of each wetland. Hydrogen ion concentration (pH), electrical conductivity (EC) and temperature (T) were measured in situ with a HACH HQ40d multiparameter device. BOD_5_ was measured with Oxytop head gas sensors after five days of incubation at 20 °C, COD was measured using the K_2_Cr_2_O_7_ method according to APHA (1998). Ammoniacal nitrogen (NH_3_—N), Nitrates (NO_3_—N) and Total Phosphorus (TP) were measured using a UVESV GENESYS 10S spectrophotometer using standard methods (APHA, 1998). Meanwhile, Fecal Coliforms (FC) were measured by membrane filter procedure. The Total Suspended Solids (TSS) were determined by the gravimetric method. Alkalinity was measured by the titration method (APHA, 1998).
Data format	Raw, summary
Parameters for data collection	Two small-scale vertical subsurface flow constructed wetland (VSSFCW) were implemented. Phragmites Australis were planted in one wetland and Cyperus Papyrus in the second wetland. Fortnightly samples were collected for three months and transferred to the laboratory for analysis. The mentioned parameters above, in abstract section, were analyzed according to the standards for water and wastewater treatment handbook.
Description of data collection	The samples were taken at an average water temperature of 24.6 °C. A residence time of the water in the wetland of 1.12 days, a flow rate of 0.6 m^3^ day^−1^ for each wetland and a hydraulic load rate (HLR) of 0.2 m day^−1^ throughout the experiment. The levels of physical-chemical and microbiological parameters of the water treated in the VSSFCWs were determined.
Data source location	Santa Isabel, Ecuador, Longitude 79.313732 °W and Latitude 3.298460 °S
Data accessibility	Data was provided in this article
Related research article	F. García-Ávila, J. Patiño-Chavez, F. Zhinín-Chimbo, S. Donoso-Moscoso, L. Flores del Pino, A. Avilés-Añazco. Performance of Phragmites Australis and Cyperus Papyrus in the treatment of municipal wastewater by vertical flow subsurface constructed wetlands. International Soil and Water Conservation Research, 7 (2019) 286–296. https://doi.org/10.1016/j.iswcr.2019.04.001

## Value of the data

• The data presented are used to analyze the treatment capacity of domestic wastewater using two species of vegetation (Phragmites Australis and Cyperus Papyrus) in vertical subsurface flow constructed wetland (VSSFCW).
• The physical-chemical and microbiological parameters data serve to provide a clear picture of the water quality that could be obtained when using Phragmites Australis and Cyperus Papyrus in VSSFCW, therefore, these data could be useful for communities or cities that have domestic wastewater with characteristics similar to this research.
• To make known that unconventional treatments in small population centers or in rural areas, is possible using VSSFCW.
• To run new experiments using these data as initial information, researchers can recognize the efficiency of Phragmites Australis and Cyperus Papyrus to develop a new set of experiments using other vegetation species and compare them with these.
• These data allow choosing a suitable vegetation for a VSSFCW that you want to implement in the treatment of domestic wastewater.

## 1 Data description

The data presented in this article refers to the quality of the treated water in two vertical subsurface flow constructed wetland (VSSFCW). Phragmites Australis was planted in a first wetland and Cyperus Papyrus was planted in a second wetland. The data obtained during the tests included parameters such as: pH, total suspended solids (TSS), electrical conductivity (CE), alkalinity (Alk), ammoniacal nitrogen (NH_3_—N), nitrate (NO_3_—N), total phosphorus (TP), biochemical oxygen demand (BOD_5_), chemical oxygen demand (COD), total coliforms (TC) and fecal coliforms (FC). The data obtained for the physicochemical and microbiological parameters of the wastewater (influent) and treated water in the VSSFCW (effluent) are shown in Table 1 (Supplemental File 1). Constructed wetlands are an emerging treatment technique that can improve the treatment of domestic wastewater [1]. The removal of contaminants is better in the presence of vegetation [2], which improves the removal efficiency of COD, BOD, TSS, NH_3_—N, TP, FC [3,4]. Based on the aforementioned, this research compared the efficiency between Phragmites Australis and Cyperus Papyrus planted in artificial underground vertical flow wetlands (VSSFCW) on a pilot scale. Summary data on water quality efficiency obtained for Phragmites Australis and Cyperus Papyrus are provided in Table 2 (Supplemental File 2).

**Table 1:** Physicochemical and microbiological parameter data of the influent; effluent data obtained in the VSSFWC with Phragmites Australis and Cyperus Papyrus during the seven monitoring.

Sampling 1 (S1). Date: 15 April	Parameters	Unit	Influent	Effluent C. Papyrus	Effluent P. Australis

	pH		6.79	6.19	5.96
	Temperature	°C	26.7	26.1	26.1
	Alkalinity	mg/L, CaCO_3_	210.2	126.6	111.6
	EC	µS/cm	680	545	521
	TSS	mg/L	55	58	22
	BOD_5_	mg/L	102.5	29.97	49.9
	COD	mg/L	205.04	89.89	78.04
	NO_3_—N	mg/L	0.605	2.105	7.615
	NH_3_—N	mg/L	22.3	3.3	2.4
	TP	mg/L	5.01	3.07	4.14
	TC	MPN/ 100ml	1.60E+10	1.60E+09	1.60E+09
	FC	MPN/ 100ml	1.60E+10	1.60E+09	1.60E+09
				
Sampling 2 (S2). Date: 29 April	Parameters	Unit	Influent	Effluent C. Papyrus	Effluent P. Australis
	pH		6.94	6.32	6.45
	Temperature	°C	23.1	22.8	23.0
	Alkalinity	mg/L, CaCO_3_			
	EC	µS/cm	772	634	665
	TSS	mg/L	78	144	82
	BOD_5_	mg/L	89.5	14.85	20.3
	COD	mg/L	280	67	99
	NO_3_—N	mg/L			
	NH_3_—N	mg/L	35.56	11.65	13.83
	TP	mg/L	7.42	3.21	3.75
	TC	MPN/ 100ml			
	FC	MPN/ 100ml			
				
Sampling 3 (S3). Date: 13 May	Parameters	Unit	Influent	Effluent C. Papyrus	Effluent P. Australis
	pH		6.95	6.3	6.2
	T emperature	°C	24.61	24.36	24.23
	Alkalinity	mg/L, CaCO_3_	175.84	68.8	62.6
	EC	µS/cm	643.25	633.66	608.5
	TSS	mg/L	88.83	60	33
	BOD_5_	mg/L	95.75	16.49	21.56
	COD	mg/L	222.44	66.01	77.35
	NO_3_—N	mg/L	0.811	7.16	8.35
	NH_3_—N	mg/L	29.52	8.94	8.65
	TP	mg/L	4.56	3.27	3.21
	TC	MPN/ 100ml	5.40E+10	1.04E+09	2.15E+09
	FC	MPN/ 100ml	1.70E+10	7.52E+08	1.07E+09
				
Sampling 4 (S4). Date: 27 May	Parameters	Unit	Influent	Effluent C. Papyrus	Effluent P. Australis
	pH		7.02	6.65	6.69
	Temperature	°C	25.4	25.5	25.3
	Alkalinity	mg/L, CaCO_3_	167	64.2	58.6
	EC	µS/cm	537	472	590
	TSS	mg/L	52	80	43
	BOD_5_	mg/L	39	16.6	20.2
	COD	mg/L	138.44	70.72	82.66
	NO_3_—N	mg/L	1.442	10.42	10.08
	NH_3_—N	mg/L	19.66	2.24	5.88
	TP	mg/L	6.4	3.14	3.21
	TC	MPN/ 100ml	1.20E+10	2.00E+08	3.30E+07
	FC	MPN/ 100ml	1.20E+10	1.70E+09	2.80E+07
Sampling 5 (S5). Date: 10 June	Parameters	Unit	Influent	Effluent C. Papyrus	Effluent P. Australis
	pH		6.97	6.25	5.91
	Temperature	°C	25.4	25.5	25.3
	Alkalinity	mg/L, CaCO_3_	199.2	17.4	18
	EC	µS/cm	602.5	1072	841
	TSS	mg/L	63	51	33
	BOD_5_	mg/L	84	14	13.6
	COD	mg/L	215.13	70.71	78.68
	NO_3_—N	mg/L	1.369	9.384	9.208
	NH_3_—N	mg/L	30.63	3.3	2.6
	TP	mg/L	10.13	3.31	4
	TC	MPN/ 100ml	3.50E+10	3.90E+08	2.10E+09
	FC	MPN/ 100ml	1.40E+10	3.90E+07	7.00E+08
				
Sampling 6 (S6). Date: 24 June	Parameters	Unit	Influent	Effluent C. Papyrus	Effluent P. Australis
	pH		6.93	5.86	5.98
	Temperature	°C	24.7	24.5	23.6
	Alkalinity	mg/L, CaCO_3_	176.8	23	62.4
	EC	µS/cm	627	548	512
	TSS	mg/L	149	8	4
	BOD_5_	mg/L	102	7.02	4.6
	COD	mg/L	235.06	54.78	66.73
	NO_3_—N	mg/L	0.33	9.96	10.13
	NH_3_—N	mg/L	38.9	20.8	11.9
	TP	mg/L	11.02	3.47	4.23
	TC	MPN/ 100ml	4.70E+10	2.00E+08	3.50E+09
	FC	MPN/ 100ml	3.90E+10	3.30E+07	2.80E+09
				
Sampling 7 (S7). Date: 8 July	Parameters	Unit	Influent	Effluent C. Papyrus	Effluent P. Australis
	pH		7.06	6.58	6.26
	Temperature	°C	22.4	21.8	22.1
	Alkalinity	mg/L, CaCO_3_	126.2	112.8	62.4
	EC	µS/cm	641	531	522
	TSS	mg/L	136	19	14
	BOD_5_	mg/L	157.5	16.5	20.8
	COD	mg/L	260	43	59
	NO_3_—N	mg/L	0.31	3.94	4.72
	NH_3_—N	mg/L	30.1	12.4	15.3
	TP	mg/L	5.01	3.64	3.91
	TC	MPN/ 100ml	1.60E+11	2.80E+09	3.50E+09
	FC	MPN/ 100ml	4.70E+09	3.90E+08	2.40E+08

**Table 2:** Average removal efficiency data during the seven tests.

Parameters	Cyperus Papyrus	Phragmites Australis
	Efficiency%	Efficiency%
Total Suspended Solids	32.46±14.7	62.85±10.14
BOD_5_	80.69±4.6	75.39±6.77
COD	69.87±4.56	64.78±4.32
Ammoniacal Nitrogen	69.69±6.17	70.70±5.62
Total Phosphorus	50.00±6.51	49.38±5.75
Total Coliforms	98.08±1.5	96.02±1.29
Fecal Coliforms	95.61±2.01	93.74±1.2

**Fig. 1:**
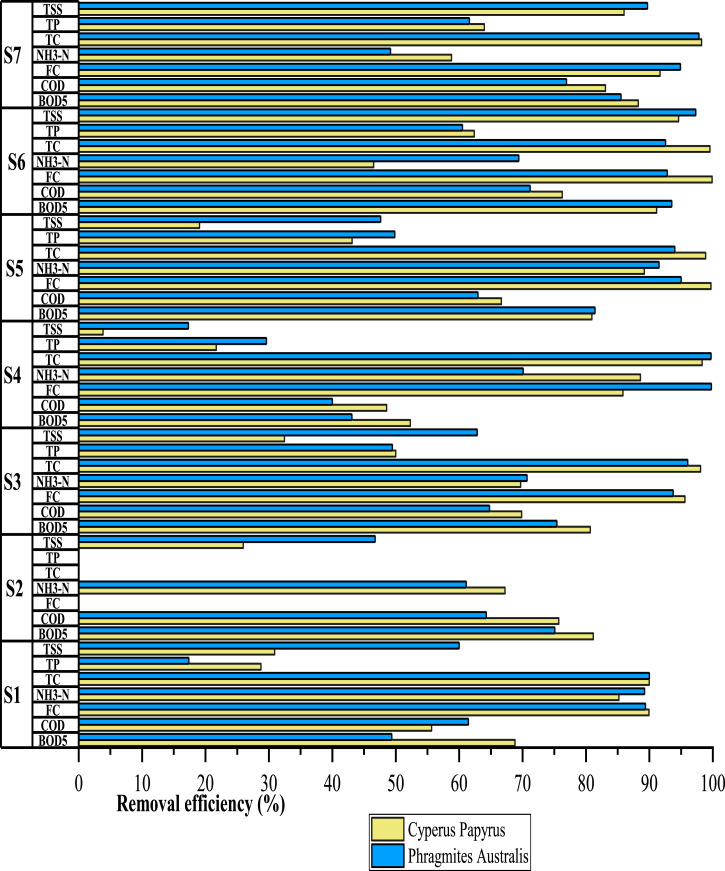
Comparison of removal efficiency between Phragmites Australis and Cyperus Papyrus in the seven monitoring.

**Fig. 2:**
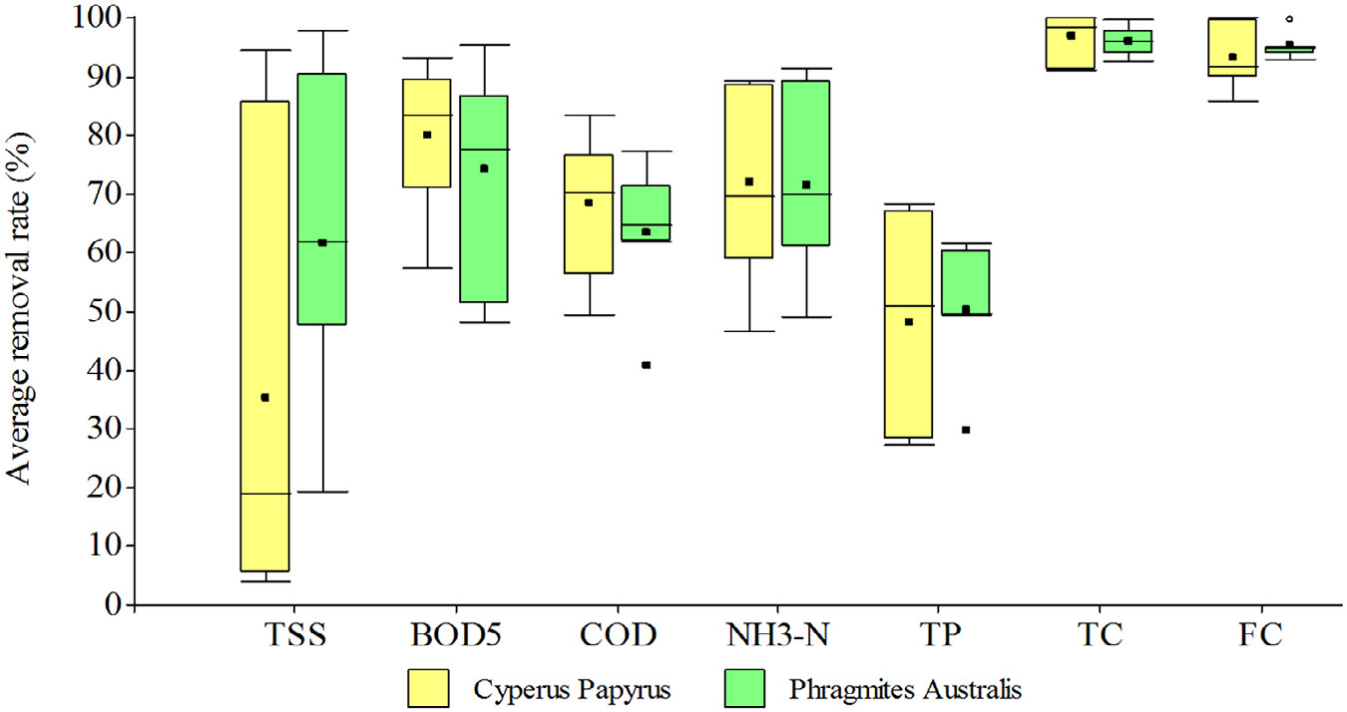
Box plot comparing average pollutant removal efficiencies using Phragmites Australis and Cyperus Papyrus.

Data presented in Table 2 refer to the average removal efficiency of TSS, BOD_5_, COD, NH_3_—N, TP, TC, FC obtained during the seven tests. Fig. 1 describes the efficiency of Phragmites Australis and Cyperus Papyrus in the removal of contaminants for each of the seven controls performed (Supplemental File 3). Fig. 2 presents a boxplot that compares the average efficiencies of parameter removal: TSS, BOD_5_, COD, NH_3_—N, TP, TC, FC. These average efficiency data refer to the three months of monitoring (Supplemental File 4).

## 2 Experimental design, materials and methods

### 2.1 Location

The VSSFCWs were implemented together with the Wastewater Treatment Plant in the city of Santa Isabel, Ecuador (Longitude 79.313732 °W and Latitude 3.298460 °S). This plant has a primary treatment through a lagoon. The effluent from this lagoon was conducted to the two VSSFCW, one for each species, Phragmites Australis and Cyperus Papyrus. Each wetland operated independently but with a continuous water flow of 0.6 m^3^ day^−1^.

### 2.2 Design and constructed of the VSSFWCs

To determine the size of the VSSFWCs, the methodology cited by García [7] was used. An initial BOD_5_ concentration of 100 mg L^−1^ (influent) and the desired BOD_5_ concentration of 10 mg L^−1^ (effluent) was considered; a water residence time of 1.12 days was determined for the system to operate continuously [5,6].

A substrate depth of 0.7 m was considered, the deeper the substrate, the greater the load that the system can process, but if the substrate is too deep, the bottom conditions become anaerobic and can reduce the removal of BOD_5_ [7]. An effective porosity of the substrate of 0.346 for sand and gravel was considered, finally obtaining an area for each VSSFWC of 3 m^2^, necessary for the reduction of BOD_5_. In Table 3, the design parameters of the pilot-scale experimental wetlands are presented. The VSSFWCs operated with an HLR of 0.2 m day^−1^. The granulometry of the material recommended by [7] was considered. The design data is described in Table 3, this table presents the design parameters of the VSSFCW, which include: HLR, Flow, Residence time, Depth, Area, Long, Width, Slope, Vegetation density (Supplemental File 5).

**Table 3:** Data of design to implement the VSSFCWs on a small scale.

Parameter	Value	Unit
HLR	0.2	m day^−1^
Flow	0.6	m^3^ day^−1^
Residence time	1.12	day
Depth	0.7	m
Area	3	m^2^
Long	3	m
Width	1	m
Slope	1	%
Vegetation density	4	plants m^−2^

### 2.3 Construction of the pilot-scale VSSFWCs

**Fig. 3:**
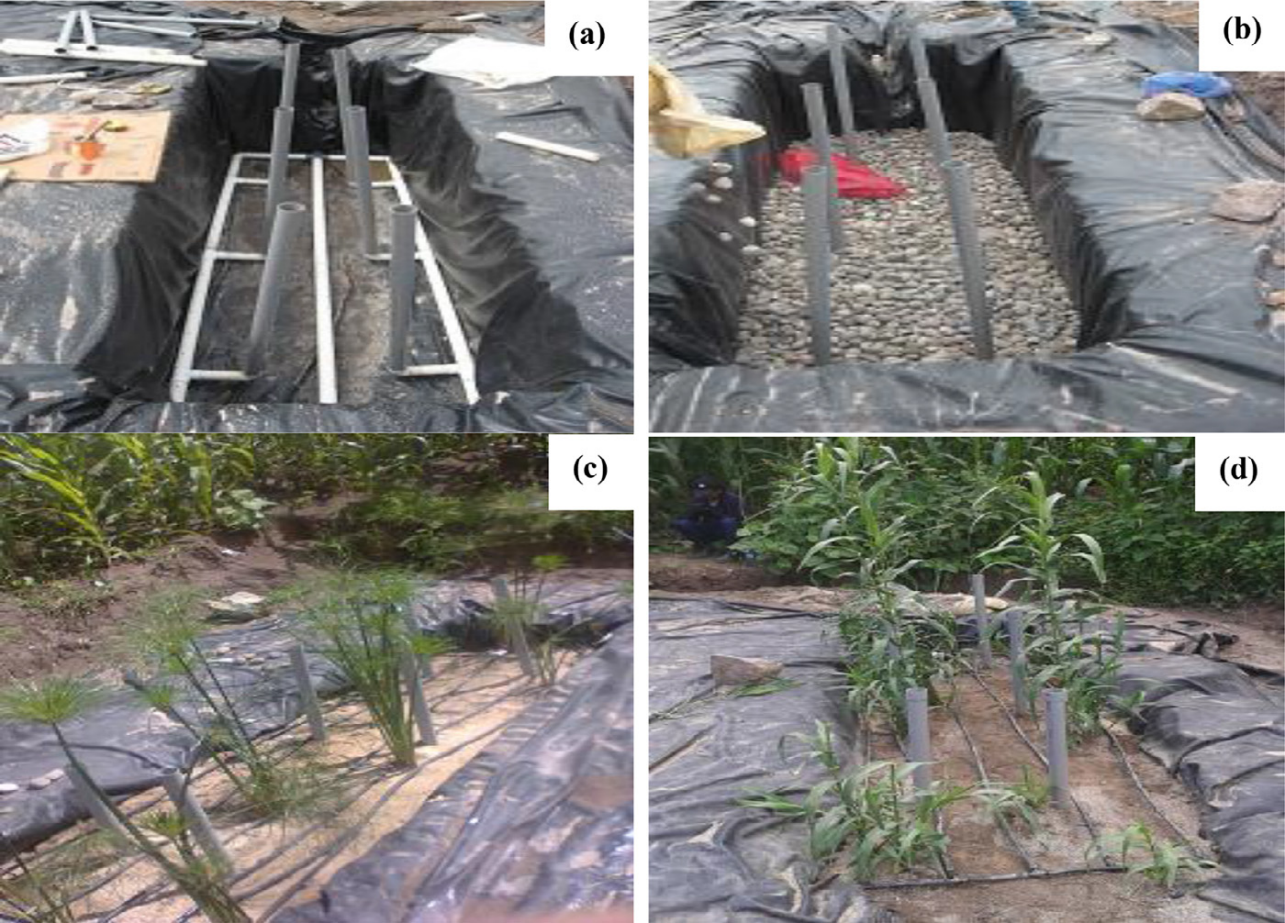
VSSFCW built on a small scale. (a) Placing drainage and aeration pipelines, (b) Assembling the filter bed, (c) VSSFCW with Cyperus Papyrus, (d) VSSFCW with Phragmites.

For the construction of the VSSFWCs, excavation work was carried out, until two pits of 3 m long, 1 m wide and 0.7 m deep each were obtained, then they were covered with high-density polyethylene (HDPE) to avoid infiltrations. The treated water drainage and conduction system were built with a 50 mm PVC pipelines and fittings. For the continuous supply of water (influent) to the VSSFWCs, 16 mm diameter polyethylene hoses were used. These hoses were drilled lengthwise to regulate flow at each point in the wetland, forming a closed-loop that allowed the influent to be distributed equally over the entire surface of the wetland. Six vertical aeration pipes connected to the drainage collection system were installed [6]. For the filter medium, gravel and silicon sand were used. Twelve plants of each investigated species (Phragmites Australis and Cyperus Papyrus) were planted for each wetland, with a density of 4 plants m^2^ (Fig. 3).

### 2.4 Collection of samples and analysis

After the Phragmites Australis and Cyperus Papyrus were planted, the wetlands were filled for a week. From the second week on, the wetland was fed water at a flow rate of 0.6 m day^−1^. During two months the development of the plants and the purified effluent were observed. After four months of planting, the plants reached a suitable height. The experiment started in January and ended in July. The sampling was carried out from April to July.

The performance efficiency of the Phragmites Australis and Cyperus Papyrus in VSSFCW was evaluated, for which water samples were collected biweekly for three months to measure: pH, total suspended solids (TSS), electrical conductivity (EC), alkalinity (Alk), ammoniacal nitrogen (NH_3_—N), nitrate (NO_3_—N), total phosphorus (TP), biochemical oxygen demand (BOD_5_), chemical oxygen demand (COD), total coliforms (TC) and fecal coliforms (FC) according to the standard methods for the examination of water and wastewater.

The data presented in this article are related to the research article entitled “Performance of Phragmites Australis and Cyperus Papyrus in the treatment of municipal wastewater by vertical flow subsurface constructed wetlands” [7].

As it can be observed in Fig. 2, the removal efficiency of BOD_5_, COD, total coliforms, fecal coliforms, ammonia nitrogen and phosphates was 80.69, 69.87, 98.08, 95.61, 69.69 and 50.0% for Cyperus Papyrus and 75.39, 64.78, 96.02, 93.74, 70.70 and 49.38% for Phragmites Australis respectively. These data allowed to determine that Cyperus Papyrus was a little more efficient compared to Phragmites Australis, therefore, Cyperus Papyrus could be a species of macrophyte ideal for wetlands built on a large scale, due to its high effect of eliminating pollutants present in wastewater domestic. These data allow choosing a suitable vegetation for a VSSFCW that you want to implement in the treatment of domestic wastewater.

## Conflict of Interest

The authors declare that they have no known competing financial interests or personal relationships that could have appeared to influence the work reported in this paper.

## Appendix Supplementary materials

Image, application 1

Appendix A. Supplementary data Image, application 2
